# A novel microdosimetry-based formalism for cell survival modelling applicable to hypofractionated radiotherapy

**DOI:** 10.1088/1361-6560/adf16d

**Published:** 2025-07-28

**Authors:** Oleg N Vassiliev, Radhe Mohan

**Affiliations:** Department of Radiation Physics, The University of Texas, MD Anderson Cancer Center, Houston, TX, United States of America

**Keywords:** proton beam therapy, hypofractionation, cell survival modelling, microdosimetry

## Abstract

**Objectives.:**

Hypofractionated radiotherapy requires reliable cell survival models for doses much higher than the standard 2 Gy, for which the linear-quadratic (LQ) model is not applicable. We developed an alternative approach applicable to both low doses and high doses used in hypofractionated treatments and radiobiological experiments.

**Approach.:**

We combined a standard microdosimetric technique with a recently introduced non-LQ cell survival model. Our formulation accounts for cell damage by multi-track events involving any number of particles. This is necessary for modelling cell survival at therapeutic doses. We characterise each cell type by the size R of the sensitive volume (SV) and biological response function B(q), where q is the total energy deposited in the SV after a given dose is delivered. q is a random quantity characterised by a probability density (microdosimetric spectrum) calculate with Geant4. We determine R and B(q) through an optimisation procedure that minimises differences between model predictions and cell survival measurements that cover an appropriate linear energy transfer (LET) range.

**Main results.:**

Our method eliminated a serious flaw of the standard microdosimetric approach—arbitrary SV size. We determine SV size by solving the above optimisation problem. Furthermore, our method drastically simplifies calculations of multi-particle microdosimetric spectra. We applied our approach to 24 proton survival curves for three cell lines with various irradiation conditions and LET range of $0.589-19.6\text{keV}\;\mu {\text{m}}^{-1}$ with good agreement between all these measurements and the model. R for a given cell type depended on fluence spectrum and increased with increasing LET owing to variations in the development and spatial spread of damage triggered by initial physical impact. This differ from the standard microdosimetry where SV size is constant.

**Significance.:**

Our model is relatively simple and suitable for implementation in a treatment planning system potentially improving treatment plan optimisation, calculation of RBEs and biologically equivalent doses.

## 1. Introduction

Hypofractionation and the use of accelerated charged particles are growing trends in radiotherapy (Durante and Formenti 2018). One of the challenges of using high doses per fraction is the well-documented limitations of the linear-quadratic (LQ) model (Elkind and Sutton 1959, Kirkpatrick *et al* 2008, Iwata *et al* 2009, Qiu *et al* 2020). For megavoltage x-rays the LQ model is ‘inappropriate’ when fractional dose is higher than 8–10 Gy (Qiu *et al* 2020). Above these doses, the survival curve (ln S vs dose) becomes a straight line. This threshold is approximate and subject to biological variability. In addition, there are no data to support these values for protons. The shape of proton survival curves depends on linear energy transfer (LET). The transition point where a curved shape that can be approximated by the LQ model becomes a straight line, which is inconsistent with the LQ dependence, shifts to lower doses when LET increases. At sufficiently high LET values this point can be at zero dose. LET distributions in proton treatment plans are rather complex. Adding to this problem, large spatial dose variations in hypofractionated treatments make the LQ model a poor choice for planning proton treatments. It needs to be replaced by a better founded model applicable to a broad range of doses, for any realistic LETs.

Several methods have been developed for improving predictions of cell survival at high doses (for example, Curtis (1986), Scholz and Kraft (1994), Guerrero and Li (2004), Carlone *et al* (2005), Beuve *et al* (2008), Park *et al* (2008), McKenna and Ahmad (2009), Guerrero and Carlone (2010), Hanin and Zaider (2010), Wang *et al* (2010), Vassiliev (2012), Zhao *et al* (2020), Li *et al* (2021)). However, these studies were largely focused on x-rays and provided only limited validation data. One notable exception is the study by Scholz and Kraft (1994). Their method became a part of the local effect model (Kramer and Scholz 2000) that has been extensively tested. The downside of that method is that it is not based on any real mechanism. Survival curves are modified essentially by hand to give them a more realistic shape.

The present study builds on a recently proposed non-LQ model (Vassiliev 2023). The model is based on two very general principles: (1) during a dose fraction delivery cell radiosensitivity increase owing to the accumulation of latent damage (i.e. all initial damage that is subject to transformation by relatively slow biological processes, and ultimately is either repaired or manifests as more permanent damage including cell death); and (2) radiosensitivity is bounded from above and stops increasing with dose when its maximum is reached. At this maximum point, the curved part of the survival curve (ln S vs dose) ends and the linear part begins. The straight line reflects inability to repair any additional damage, which means a predominantly single-hit cell kill mechanism. For this reason, the slope of the line is determined by a single parameter: the frequency average specific energy $z_{\text{F}}$ that strongly depends on sensitive volume (SV) size. Vassiliev (2023) reviewed properties of the non-LQ model and evaluated its performance using hundreds of survival curves, for protons, x-rays and heavy ions. A particularly useful aspect of the model is that its mathematical formulation is perfectly compatible with the microdosimetric formalism, as we demonstrate in the next section.

We applied our approach to a set of 24 proton survival curves for three cell lines with different radiosensitivities: AG01522, U87 and V79 from four sources: Blomquist *et al* (1993), Robertson (1994), Chaudhary *et al* (2014), Wouters *et al* (2015). The data cover an LET range of $0.589-19.6\text{keV}\;\mu {\text{m}}^{-1}$. Out of abundant data for V79 cells we chose 12 experiments that provide almost uniform LET sampling over the range: $0.589-5.59\text{keV}\;\mu {\text{m}}^{-1}$, which is most relevant for treatment planning. Using data from multiple sources and representing a variety of beam properties, helps us reduce the risk of systematic errors due to selection bias and allows us to see the magnitude of real uncertainties. We present step by step our optimisation procedure, which is substantially different from the standard microdosimetric approach to survival modelling. Then we review the properties of model parameters and outline how our method can be implemented in a treatment planning system.

## 2. Methods

### 2.1. Combining microdosimetry with the non-LQ cell survival model

The purpose of the study by Vassiliev (2023) was to explain at a fundamental level the general shape of cell survival curves ln S versus dose, in particular, the transition to linear dependence at high doses. That study also provided a formula for cell survival based on the single-target multi-hit concept. This formula produces the correct survival shape at high doses. The formula is also accurate in the low-dose limit. The first two nonzero terms in the power series expansion in dose coincide with the LQ formula. An innovative aspect of that study is that for a given cell type the SV size is not fixed. It depends on beam quality, because in this approach, SV size characterises the range of interaction between damaged DNA sites. Thereby it accounts for the spread of damage around the particle path by physical, chemical and biological processes triggered by the initial radiation impact. SV size increases with increasing LET. This conclusion was based on a review of extensive experimental data. The same trend was also observed for inactivation cross sections.

A note on the terminology: in microdosimetry, the term ‘site’ is often used instead of ‘target volume’; we will use the term ‘sensitive volume’ (SV) for consistency with our previous work.

To model the LET, or more generally beam quality, dependence of cell survival we employ the formalism of microdosimetry. We start with a standard technique (ICRU 1983, Vassiliev 2017). Then, we combine it with the non-LQ formula (Vassiliev 2023). This results in substantial improvements compared with the standard microdosimetry. In microdosimetry, radiation impact after a dose D is delivered is characterised by the probability density f(q∣D) of the total energy q deposited in the SV by all particles. It is normalised to 1 excluding the singular point q=0:

(1) $$\int_{0+}^{\infty }f(q|D)\text{d}q=1.$$

Then we introduce the biological response function B(q) that maps distribution f(q∣D) onto cell survival

(2) $$S(D)=\int_{0+}^{\infty }B(q)f(q|D)\text{d}q.$$

B(q) is the probability of a cell surviving energy q deposited in SV. B(q) is undefined for q<0. Elsewhere, it is bounded 0⩽B(q)⩽1, nonincreasing dB/dq⩽0, and satisfies B(0)=1.

Equation (2) is the basis of the standard microdosimetric approach to modelling cell survival. To model cell survival for a given cell type, an optimisation procedure is executed that finds such B(q) that minimises differences between a set of survival curves (left side, equation (3)) and model predictions (right side):

(3) $$S_{k}\left(D_{kj}\right)=\int_{0+}^{\infty }B\left(q\right)f\left(q|D_{kj}\right)\text{d}q;\;k=1,2,\ldots ,m;\;j=1,2,\ldots n_{k}.$$

In this equation, $S_{k}$ is kth survival curve in a cell survival data set. The total number of survival curves in the data set is m. Each survival curve is a set of measurements at doses $D_{kj}$, where k is the curve number and j is the number of a dose point. The total number of dose points in kth curve is $n_{k}$.

The above approach has two serious problems. First, distribution f(q∣D) strongly depends on the size of SV. However, ‘there is no clear indication of the relevant site size for specific biological endpoints’ NCRP 2018, 181. Second, $f\left(q|D_{ij}\right)$ needs to be calculated for each dose in the set $\left\{D_{kj}\right\}$. These calculations must account for the fact that the number of particles that contribute to the total deposited energy q is random in interval 0 to ∞. Such calculations are time consuming. We eliminate both these problems by applying equations (2) and (3) to the non-LQ cell survival formula proposed by Vassiliev (2023). The formula is simple and most general

(4) $$S\left(D\right)={\text{e}}^{-N}\sum_{i=0}^{\infty }\frac{N^{i}}{i!}s_{i},$$

where

(5) $$N=\frac{D}{z_{\text{F}}},$$

is the average number of hits at a given dose, $z_{\text{F}}$ is the frequency average specific energy, and $s_{i}$ is the probability of a cell surviving i hits (i.e. energy deposition events). $s_{i}$ characterise the ability of a cell to repair damage. Obviously, $s_{0}=1$ and we can expect that $s_{i+1}⩽s_{i}$.

We can also represent f(q∣D) by a similar sum (Vassiliev 2017):

(6) $$f(q|D)={\text{e}}^{-N}\sum_{i=0}^{\infty }\frac{N^{i}}{i!}f_{i}(q).$$

The above is simply the total probability formula, in which we assumed that the number of hits is Poisson-distributed. Here $f_{i}(q)$ is a conditional distribution of q. The condition is that the number of hits is i. All $f_{i}(q)$ have the same normalisation as f(q∣D), equation (1). Next, we use equation (4) in the left side of equation (2) and use equation (6) in the right side:

(7) $$\sum_{i=0}^{\infty }\frac{N^{i}}{i!}s_{i}=\sum_{i=0}^{\infty }\frac{N^{i}}{i!}\int_{0+}^{\infty }B(q)f_{i}(q)\text{d}q.$$

This equation must be satisfied for any N. Therefore

(8) $$s_{i}=\int_{0+}^{\infty }B(q)f_{i}(q)\text{d}q;\;i=1,2,\ldots ;\;s_{0}=1.$$

Equations (4) and (8) drastically simplify the optimisation problem. Equation (3) is now replaced with

(9) $$S_{k}\left(D_{kj}\right)=\text{exp}\left(-\frac{D_{kj}}{z_{Fk}}\right)\sum_{i=0}^{\infty }{\left(\frac{D_{kj}}{z_{Fk}}\right)}^{i}\frac{s_{i}}{i!};\;k=1,2,\ldots ,m;\;j=1,2,\ldots n_{k}.$$

where $s_{i}$ is given by equation (8). The first simplification is that $s_{i}$ does not depend on dose, it is a function of the number of hits, i. Second, for all proton cell survival curves analysed in the present study, we can set $s_{i}=0$ for i>4 or i>2 depending on the cell type. This means that instead of the dose-dependent distribution f(q∣D) we need to calculate no more than four dose-independent distributions $f_{1}(q),f_{2}(q),f_{3}(q),f_{4}(q)$. The most important improvement is that, in contrast to standard microdosimetry, in our method SV size is not chosen arbitrarily, it is now a fitting parameter. This is because $z_{\text{F}}$ strongly depends on SV size, R:

(10) $$z_{\text{F}}=\frac{y_{\text{F}}}{\pi \rho R^{2}}\approx \frac{{\text{LET}}_{\text{F}}}{\pi \rho R^{2}},$$

where $y_{\text{F}}$ is the frequency average lineal energy, LET_F_ is the frequency average LET, and ρ is the mass density of material within SV. $y_{\text{F}}$ can be approximated by LET_F_ when R>rsim100nm (Vassiliev *et al* 2019). Variable SV size increases the amount of calculations, because distributions $f_{i}$ has to be calculated for each size. These variations, however, are a real effect that must be accounted for. This effect was overlooked in the standard microdosimetry.

### 2.2. Microdosimetric Spectra

This study is limited to protons. We also consider photon data as a reference radiation. For photons we use a method that does not require microdosimetric spectra. For protons, first, we calculate the single-event spectrum, $f_{1}(q)$, using Geant4 and a Monte Carlo algorithm previously described and validated (Vassiliev *et al* 2019). The algorithm accounts for the nanoscale dose buildup and indirect events, in which a proton misses SV but secondary electrons reach it and deposit energy. Next, using $f_{1}(q)$ as input, we calculate multi-event spectra $f_{i}(q),i>1$ by numerical integration, according to the recurrence relation (Vassiliev 2017):

(11) $$f_{i+1}\left(q\right)=\int_{0+}^{q}f_{i}\left(q^{\prime }\right)f_{1}\left(q-q^{\prime }\right)\text{d}q^{\prime }.$$

Energy distribution of protons entering an SV corresponding to an experimental survival curve $S_{k}\left(D_{kj}\right)$ is given by the fluence spectrum $\Phi_{i}(E)$. Fluence spectra are rarely reported in experimental papers. Usually, only experimental setup, basic beam parameters, and depth-doses are reported. We used this information to setup Monte Carlo calculations of all $\Phi_{i}(E)$. We used TOPAS (Perl *et al* 2012, Faddegon *et al* 2020) for these calculations. We adjusted our setup parameters, including those of the range modulation wheel, until agreement between Monte Carlo and reported depth-doses was achieved. These adjustments were performed using optimisation software. Given the large number of adjustable parameters, we were able to reproduces all the experimental depth-doses with good accuracy. Using best-fit values of the parameters, we generated fluence spectra at each depth at which survival curves were measured.

### 2.3. Optimisation methods

For implementation of our formalism into a treatment planning system, in addition to the distribution of physical dose $D(\vec{r})$, beam quality needs to be characterised at every point $\vec{r}$ of the irradiated volume. For this purpose we chose fluence spectrum, $\Phi (\vec{r},E)$. This fundamental quantity can be found by solving the Boltzmann transport equation by Monte Carlo or a deterministic method. If $\Phi (\vec{r},E)$ is known, many other beam quality characteristics can be found: average LET, LET spectra, microdosimetric spectra, and others.

#### 2.3.1. Specific energy $z_{F}$ and SV radius, R

To determine $z_{\text{F}}$ we develop a regression model and find its parameters using an optimisation procedure. $z_{\text{F}}$ is represented as an integral of weighted normalised fluence spectrum

(12) $$z_{\text{F}}={\left[\int_{0}^{\infty }\Phi (E)B_{z}(E)\text{d}E/\int_{0}^{\infty }\Phi (E)\text{d}E\right]}^{-1}.$$

The weighting function $B_{z}(E)$ is approximated by a third order polynomial of 1/E.

(13) $$B_{z}(E)=\sum_{l=1}^{4}\frac{p_{l}}{E^{l-1}}.$$

We chose this particular form based on a review of the relevant data.

To account for variable SV size we employ the following five-step optimisation procedure.

1. All survival curves for a given cell type are fit separately using equation (9). The fitting parameters are: $z_{\text{Fk}}$ and $s_{1},s_{2}\ldots$. Microdosimetric spectra are not used at this step. The number of parameters $s_{i}$ is infinite. However, we found that it suffices to fit only a subset of 2–3 consecutive $s_{i}$. All other $s_{i}$ are set to 1 (small i) or to 0 (large i). If $s_{i}=0$ or $s_{i}=1$, then we do not need to calculate the corresponding $f_{i}(q)$ at any further steps. We fit each survival curve several times using different $s_{i}$ subsets, until we identify one that provides best fit. In half of all cases in our proton data set $\left(s_{1},s_{2}\right)$ is the optimal choice. *All* $0<s_{i}<1$ *are identified, and the first iteration of* $z_{\text{Fk}}$ *set is generated*.
2. We use the set of best-fit $z_{\text{Fk}}$ to determine parameters $p_{1}$ of the regression model for $z_{\text{F}}$, equations (12) and (13). We need this model, because it allows us to calculate $z_{\text{F}}$ for an arbitrary proton beam, Φ(E). Then, for each experimental survival curve, we generate a new $z_{\text{Fk}}$, now using the regression model. *The first iteration of the regression model and the second iteration of* $z_{\text{Fk}}$ *set are generated*.
3. For each updated $z_{\text{Fk}}$ we determine SV size $R_{k}$. We cannot use for that equation (10), because $y_{\text{F}}$ is unknown. Instead, we use the try-and-error method. We pick a reasonable $R_{k}$, then calculate with Geant4 $f_{1}(q)$ and the corresponding $z_{\text{Fk}}$. Then we adjust $R_{k}$ using the $1/R^{2}$ dependence given by equation (10) until Monte Carlo $z_{\text{Fk}}$ agrees within ±1% with $z_{\text{Fk}}$ predicted by the regression model. The $1/R^{2}$ dependence is quite accurate, so it usually takes only 2–3 iterations to determine $R_{k}$. After completing this step we will also have calculated all $f_{1}(q)$ for all correct sizes $R_{k}$. *All* $R_{k}$ *for the current iteration of* $z_{\text{Fk}}$ *set are determine and corresponding* $f_{i}(q)$ *are calculate. We now have the initial state for global optimisation*.
4. First global optimisation. We fit simultaneously all survival curves for a given cell type. We optimise parameters $p_{l}$ and biological response function B(q). We discuss the latter part in more detail in the next section. Re-optimisation of $p_{l}$ produce new set of $z_{\text{Fk}}$. Then, as described above, we determine new $R_{k}$ and a new set of distributions $f_{1}(q)$. *Final iterations for the regression model and* $z_{\text{Fk}}$ *set are derived*; $R_{k}$ *and* $f_{i}(q)$ *are updated accordingly; the first iteration of* B(q) *is derived*.
5. Final global optimisation. This step is similar to step 4, except now only B(q) is optimised. All other parameters remain fixed. *The final iteration of* B(q) *is produced*.

#### 2.3.2. The biological response function, B(q)

For each cell line we determine a unique function B(q). Our procedure is based on equation (9). Simultaneous fitting of multiple cell survival curves poses two challenges. First, low-dose points contribute much more to standard loss functions, such as the sum of squared residuals, than do high-dose points. Second, there are large variations in uncertainties between individual data points. The common practice of weighting each point by inverse standard error is wrong. Uncertainties of estimated errors are higher than those of the survival data. Also, the estimated errors are random numbers, and dividing by random numbers usually results in bad statistical properties. In short, with this weighting, the optimiser is largely driven by noise.

To achieve a better balance of contributions to the loss function L from high- and low-dose points, we fit ln S instead of S. If survival was exponential, this method would produce the standard linear regression. To mitigate the influence of large variations in uncertainties of survival data, we calculate for each survival curve the Huber loss $H_{k}$ (Huber, 1964). In this method, based on statistics of residuals, points are identified that are likely to have a high uncertainty, and contributions from such points to $H_{k}$ are reduced. For fitting simultaneously multiple survival curves, we minimise the following loss function

(14) $$L=\sum_{k=1}^{m}H_{k}.$$

The biological response function B(q) must have certain properties that we listed previously. A properly scaled hyperbolic tangent satisfies this requirement. Hence, we chose the following form for B(q):

(15) $$B(q)=a{\left[\frac{1}{2}\text{tanh}\left(-\frac{q-q_{0}}{b}\right)+\frac{1}{2}\right]}^{c}.$$

This model has three independent parameters: b,c, and $q_{0}$. Parameter a can be found from the condition B(0)=1:

(16) $$a={\left[\frac{2}{\text{tanh}\left(q_{0}/b\right)+1}\right]}^{c}.$$

### 2.4. Implementation into a treatment planning system

It is impractical to use microdosimetric spectra in treatment planning software. Fortunately, there is a method for replacing spectra $f_{i}(q)$ with fluence spectra Φ(E). The method requires a few pre-tabulated 2D functions that map the (E,R) space onto (q,R). For an explanation of the algebra, we refer to Vassiliev *et al* (2019). First, we need to introduce microdosimetric spectra normalised per one source particle, $f_{i}(q|1)$. These functions have a singularity at q=0, because the probability of a source particle missing SV entirely is a finite number. We also introduce the probability of a non-zero energy deposit f(q>0∣1). From the formula for conditional probability, we can write:

(17) $$f_{i}\left(q\right)=\frac{f_{i}\left(q|1\right)}{f\left(q>0|1\right)}.$$

Further, for polyenergetic beams, we have

(18) $$f_{i}\left(q|1\right)=\int_{0}^{\infty }\Phi \left(E\right)f_{i}\left(q|1,E\right)\text{d}E,$$

and a similar formula is valid for f(q>0∣1). Next, using the above expressions, we transform equation (8):

(19) $$s_{i}=\frac{\int_{0}^{\infty }\Phi (E)B_{i}(E)\text{d}E}{\int_{0}^{\infty }\Phi (E)f(q>0|1,E)\text{d}E},$$

where $B_{i}(E)$ is a biological response function that depends on proton energy

(20) $$B_{i}\left(E\right)=\int_{0+}^{\infty }B\left(q\right)f_{i}\left(q|1,E\right)\text{d}q.$$

Then, to implement our method in treatment planning software, a small number of these functions, for example, $B_{1}(E),B_{2}(E),B_{3}(E)$ and $B_{4}(E)$ need to be tabulated. These will be two-dimensional tables, because $B_{i}(E)$ also depend on R. With these tables probabilities $s_{i}$ are calculated as a simple one-dimensional integral. Generating tables of $B_{i}(E)$ involves extensive Monte Carlo simulations. However, for any given cell type represented by a B(q), these calculations are performed only once.

We will also need to determine R for any given fluence spectrum. First, we determine $z_{\text{F}}$ using the regression model. Then, for a given $z_{\text{F}}$ we solve this equation for R

(21) $$z_{\text{F}}=\frac{y_{\text{F}}(R)}{\pi \rho R^{2}}.$$

This requires pretabulated $y_{\text{F}}$ derived using the method we used above for $f_{i}(q)$.

## 3. Results and discussion

### 3.1. Cell survival data-set

All cell survival data analysed in the present study are listed in table 1. Chaudhary *et al* (2014) results were chosen because they extend to high LET values. We also chose V79 cells, because abundant data are available. We were able to select 12 experiments that almost uniformly sample an important for treatment planning LET range: $0.589-5.59\text{keV}\;\mu {\text{m}}^{-1}$. Throughout the present study ‘LET’ means frequency average LET. This is for the following reasons: (1) consistency with the microdosimetric terminology; (2) ‘track average LET’, when it was first introduced, meant initial kinetic energy of a heavy charged particle divided by its total range, and this is not what we and many recent studies calculate; (3) dose average quantities do not appear anywhere in our formalism; (4) frequency average LET has a good predictive power whereas dose averaging, if done correctly, can produce value strongly contradicting relative biological effectiveness (RBE) data (Vassiliev 2021).

### 3.2. Reference radiation

**Figure 1.**
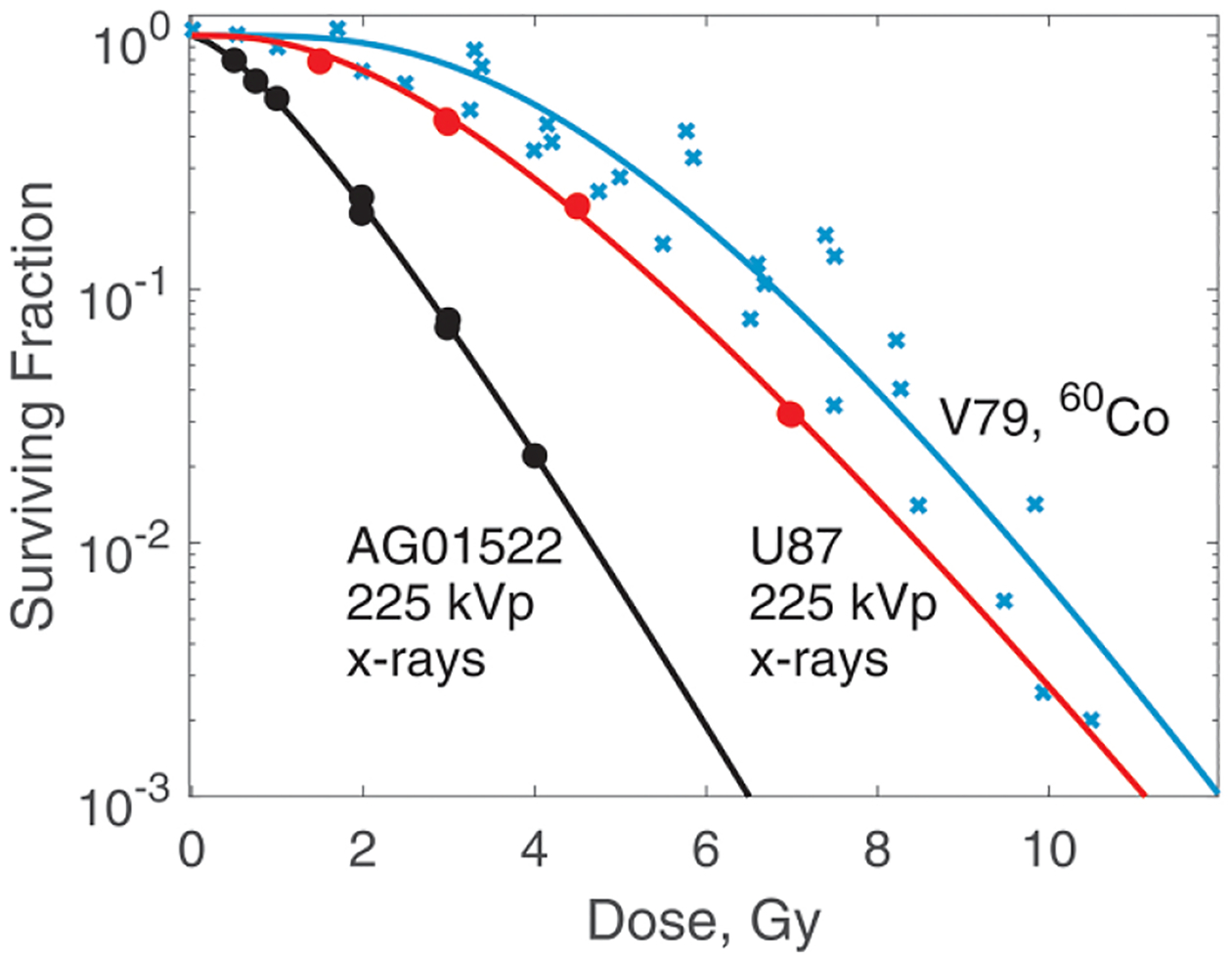
Survival of AG01522, U87 and V79 cells irradiated with 225 kVp x-rays and ^60^Co γ radiation. The solid lines show the model, the circles show experimental data from Chaudhary *et al* (2014), and the crosses show experimental data from Blomquist *et al* (1993), Wouters *et al* (2015).

The reference radiation for AG01522 and U87 cells was 225 kVp x-rays. For all V79 cells measurements, the reference radiation was ^60^Co. A simple formula is available (Vassiliev *et al* 2020) for the conversion of RBE values from one reference photon radiation to another. Here, we did not do this conversion. We used equation (9) to fit these data. The fitting parameters were $z_{F},s_{i}$ and survival at zero dose, $S_{0}$. $S_{0}$ was not constrained to be 1 in order to account for uncertainties in plating efficiency. However, it was bounded within ±0.05. We do not have sufficient data to determine B(q) for photons. Hence, we did not use microdosimetric spectra in this case. Figure 1 shows results of the fit. The significant scatter of V79 data points reflects discrepancies between data from different sources. The best fit values of all the parameters are given in table 2. Two observations can be made. First, most cells survive single hit, even when irradiated by 225 kVp x-rays with an ${\text{LET}}_{\text{F}}=2.0\text{keV}\;\mu {\text{m}}^{-1}$ (Vassiliev *et al* 2020). Second, radiosensitivity is a result of the interplay between $z_{\text{F}}$, which is related to the range of interaction between damaged sites, and $s_{i}$ that characterise the ability of a cell to repair damage.

To apply our cell survival model to photons, B(q) are not necessary. It is easier for each beam energy, for example 6 MV, 6 MV FFF, 10 MV, etc determine three parameters, $z_{\text{F}}$ and two s values, as table 2 shows.

### 3.3. Microdosimetric spectra

**Figure 2.**
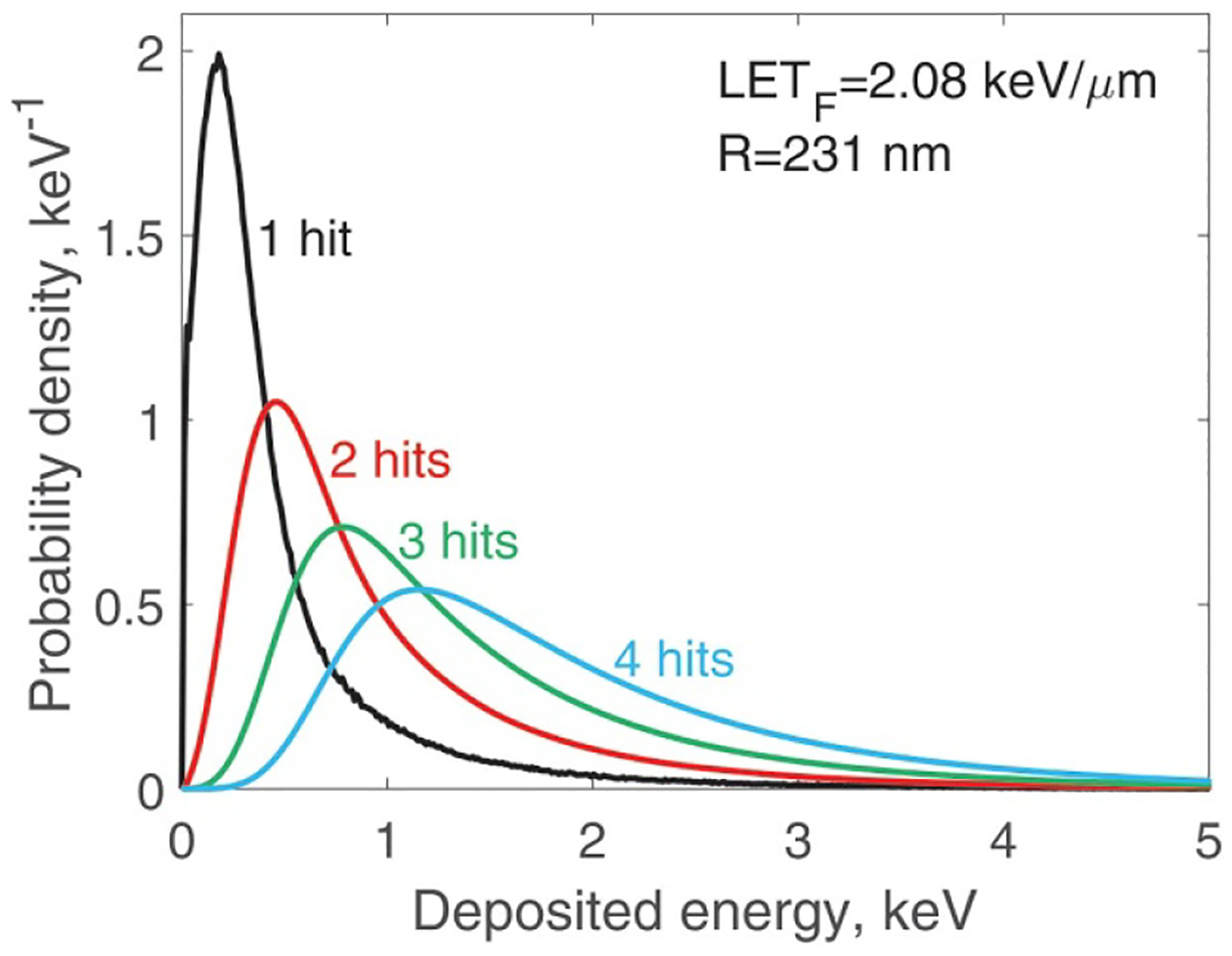
Microdosimetric spectra $f_{1}(q),f_{2}(q),f_{3}(q)$, and $f_{4}(q)$ for an example case used in modelling V79 cells survival.

Calculation of $f_{1}(q)$ spectra with Geant4 is the most time consuming part of our approach. For each of the 24 survival curves, these calculations are repeated about five times using different SV sizes. Transforming an $f_{1}(q)$ spectrum into a multi-particle spectra $f_{i}(q),i>1$, takes a few seconds. This transformation includes integration that reduces statistical uncertainties. All microdosimetric spectra in the present study are for polyenergetic proton beams. Figure 2 shows an example of calculated spectra from one of the cases used in the present study.

### 3.4. $z_{\text{F}}$ and SV size R

**Figure 3.**
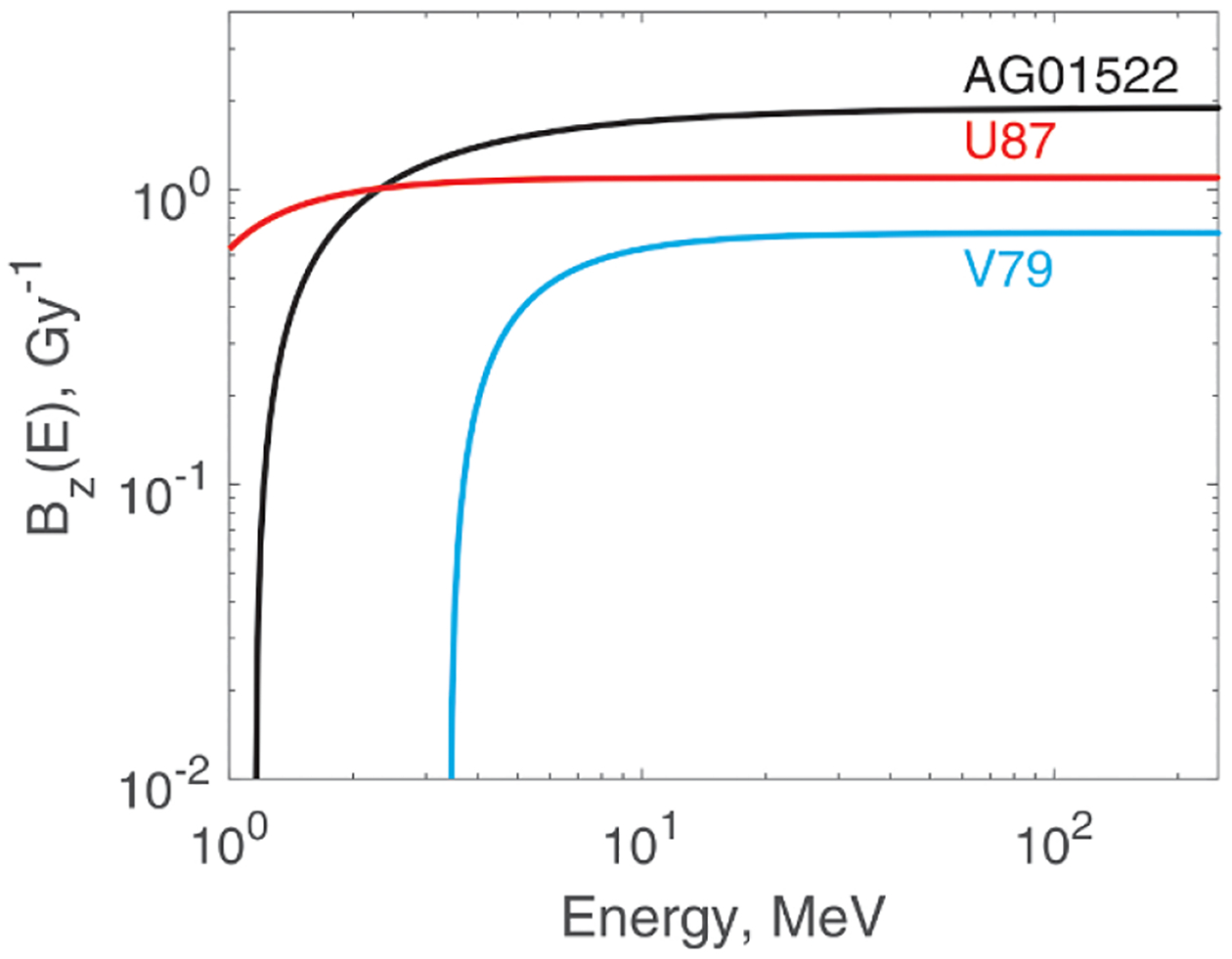
Functions $B_{z}(E)$ defined in equations (12) and (13) for the three cell lines. Higher values of $B_{z}(E)$ result in smaller $z_{\text{F}}$ and larger SV size R.

Table 3 presents best-fit values of parameters $p_{1}$ of the regression model for $z_{\text{F}}$. The corresponding weighting functions $B_{z}(E)$ are plotted in figure 3. The logarithmic scale was chosen for a better view of the most variable parts of the curves.

**Figure 4.**
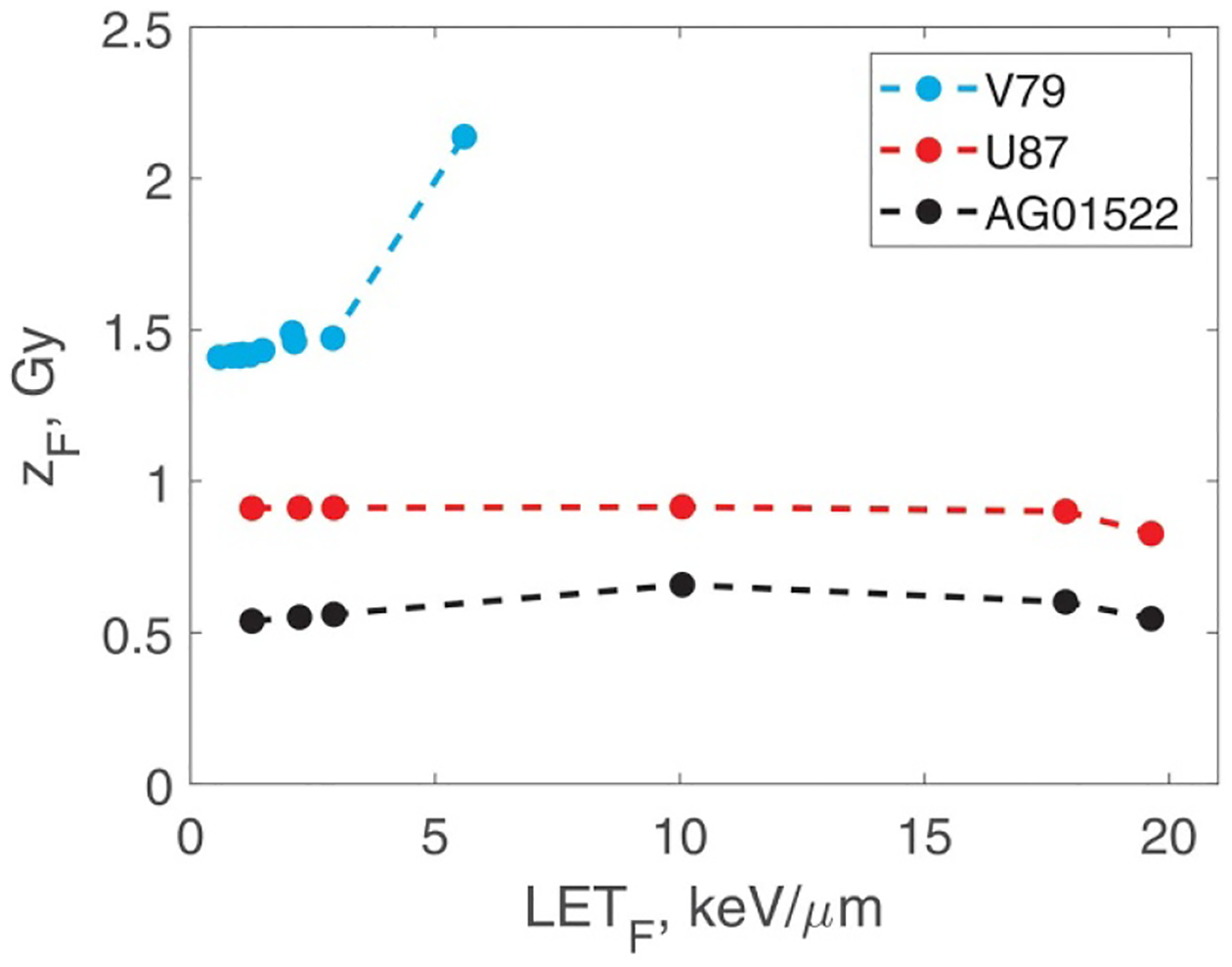
$z_{\text{F}}$ versus LET_F_ for the three cell lines. The average number of hits at a given dose is $D/z_{\text{F}}$.

Figure 4 shows $z_{\text{F}}$ as a function of ${\text{LET}}_{\text{F}}$ for the three cell lines. $z_{\text{F}}$ determines cell survival at high doses. The asymptotic expression for all cell survival curves in the high-dose limit was derived previously (Vassiliev 2023). The result is that ln S is a linear function of dose with the slope $1/z_{\text{F}}$:

(22) $$S\to \text{exp}\left(-\frac{D}{z_{\text{F}}}\right).$$

**Figure 5.**
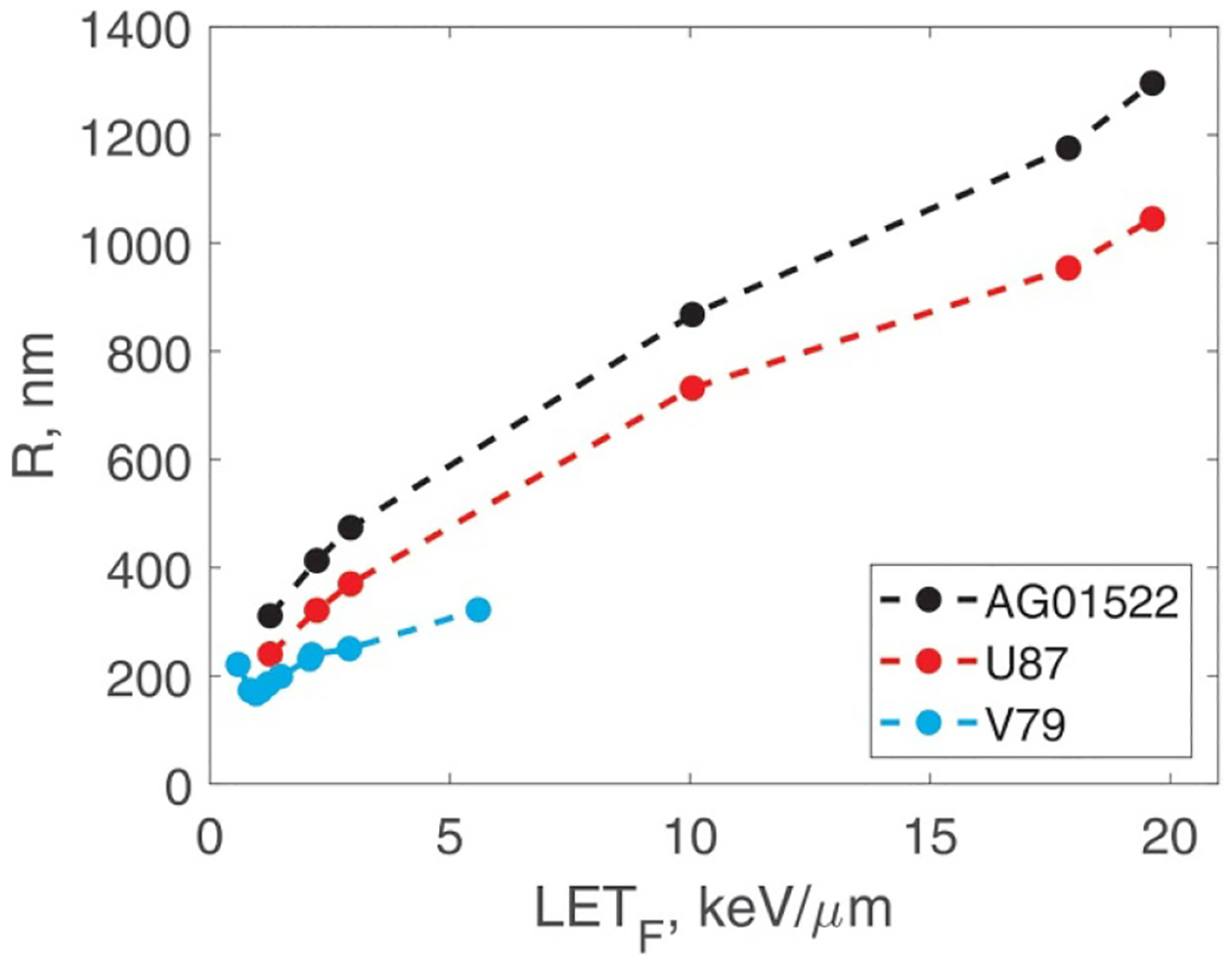
Radius of the sensitive volume, R, versus LET_F_ for the three cell lines. Radiosensitivity at high doses tends to be higher for cells with a larger R.

This means that a larger $z_{\text{F}}$ value (and therefore a smaller R value) indicate higher radioresistance at high doses. The corresponding SV sizes are shown in figure 5. For all three cell lines, R tends to increase with increasing LET_F_. This trend and the numerical values are consistent with our previous study and with the behaviour of inactivation cross sections (Vassiliev 2023). R is a single biological parameter that determines the slope of cell survival curves at high doses. The slope depends strongly on R. From equations (21) and (22), $\text{slope}\propto R^{2}/y_{\text{F}}(R)$. For $R≳100\;\text{nm},y_{\text{F}}$ depends only weakly on R and, in fact, can be approximated by LET_F_ Vassiliev *et al* (2019). Hence, $\text{slope}\propto R^{2}$. These observations emphasise the high importance of variable SV size that our model has introduced.

### 3.5. Biological response function, B(q)

**Figure 6.**
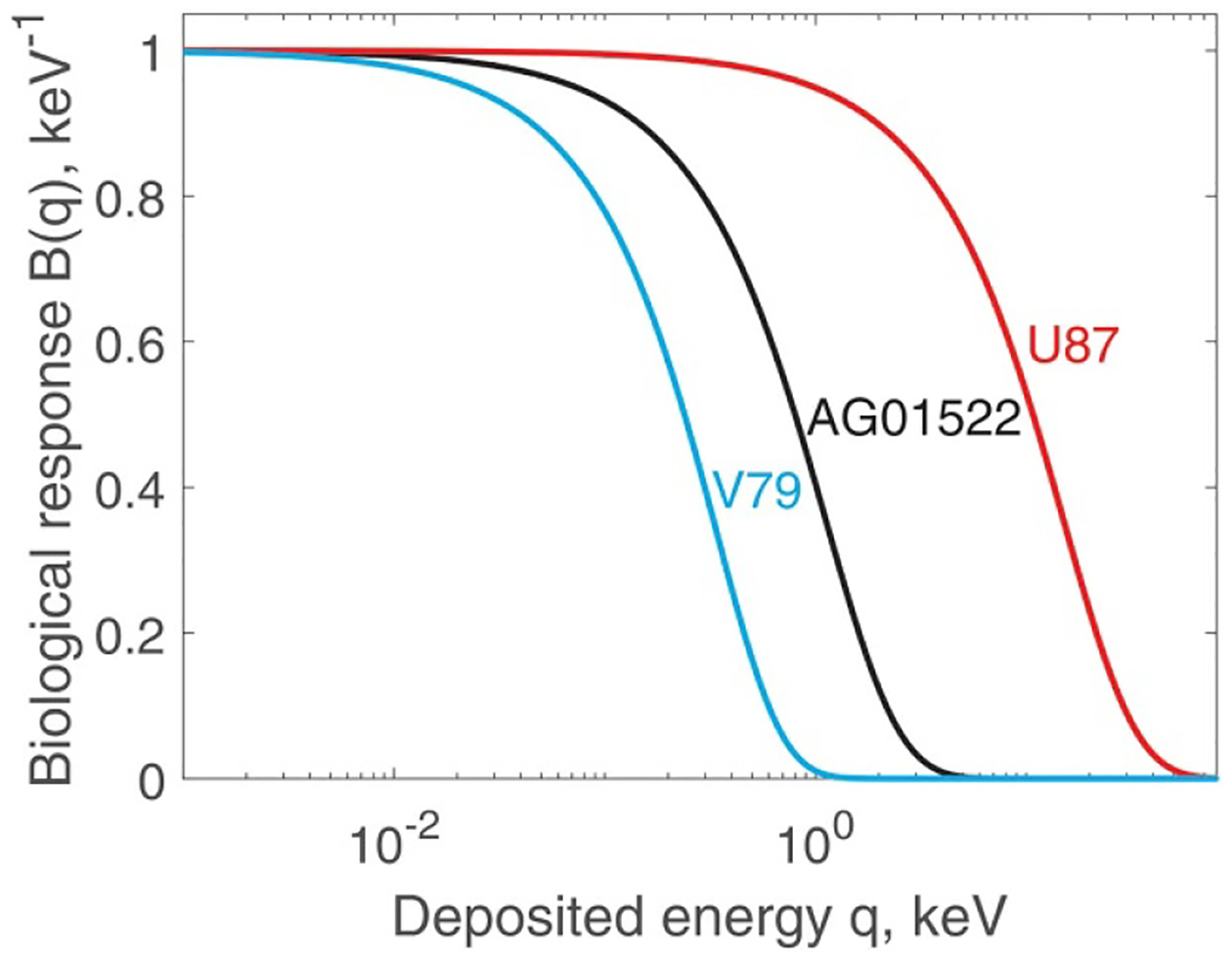
Biological response function B(q) defined in equations (2) and (15), for three cell lines.

Best-fit values of all the parameters that define B(q) are given in table 4. These functions for the three cell types are plotted in figure 6. They all originate at B(0)=1. Then, they have an almost flat part indicating that most cells will survive q<0.1keV (i.e. three ionisations). Then, a steep, approximately exponential, decrease begins. The interpretation of figure 6. is complicated by the fact that B(q) alone does not determine $s_{i}$. Instead, we need to consider the product $B(q)f_{i}(q)$, according to equation (8), where $f_{i}(q)$ depends strongly on SV size, R. For illustration purposes only, let us assume that R(U87)=R(AG01522). Then, figure 6 suggests that U87 cells are more radioresistant at low doses than AG01522. Further, we assume that R(V79)=R(U87)/2. Assuming that all cells receive the same dose, this means that the average energy deposited in SV(V79) is 1/8 of that deposited in SV(U87). This difference is reflected in figure 6 by a substantial shift of the V79 curve towards lower energies. The reason for the shift is that $f_{i}(q)$ for V79 does not reach energies q as high as those reached by $f_{i}(q)$ for VU87.

### 3.6. Cell survival and RBE

**Figure 7.**
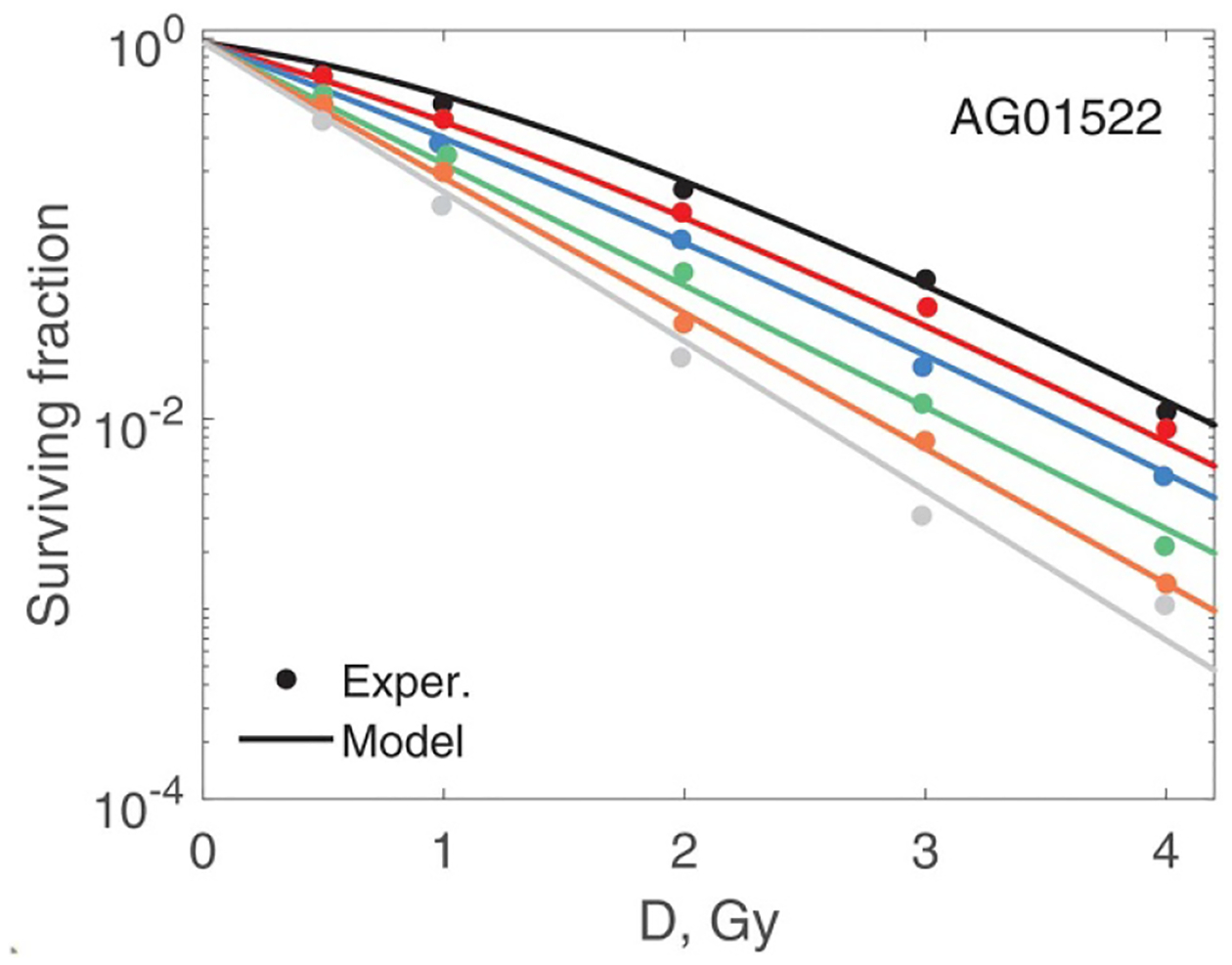
Modelling of AG01522 cell survival (Chaudhary *et al* 2014). Experiments 1–6 from table 1 are shown from top to bottom. Circles and lines of the same colour represent the same experiment. The overall r.m.s. error is 0.15 and adjusted $r^{2}$ is 0.99. Adapted from Chaudhary *et al* (2014), Copyright (2014), with permission from Elsevier.

**Figure 8.**
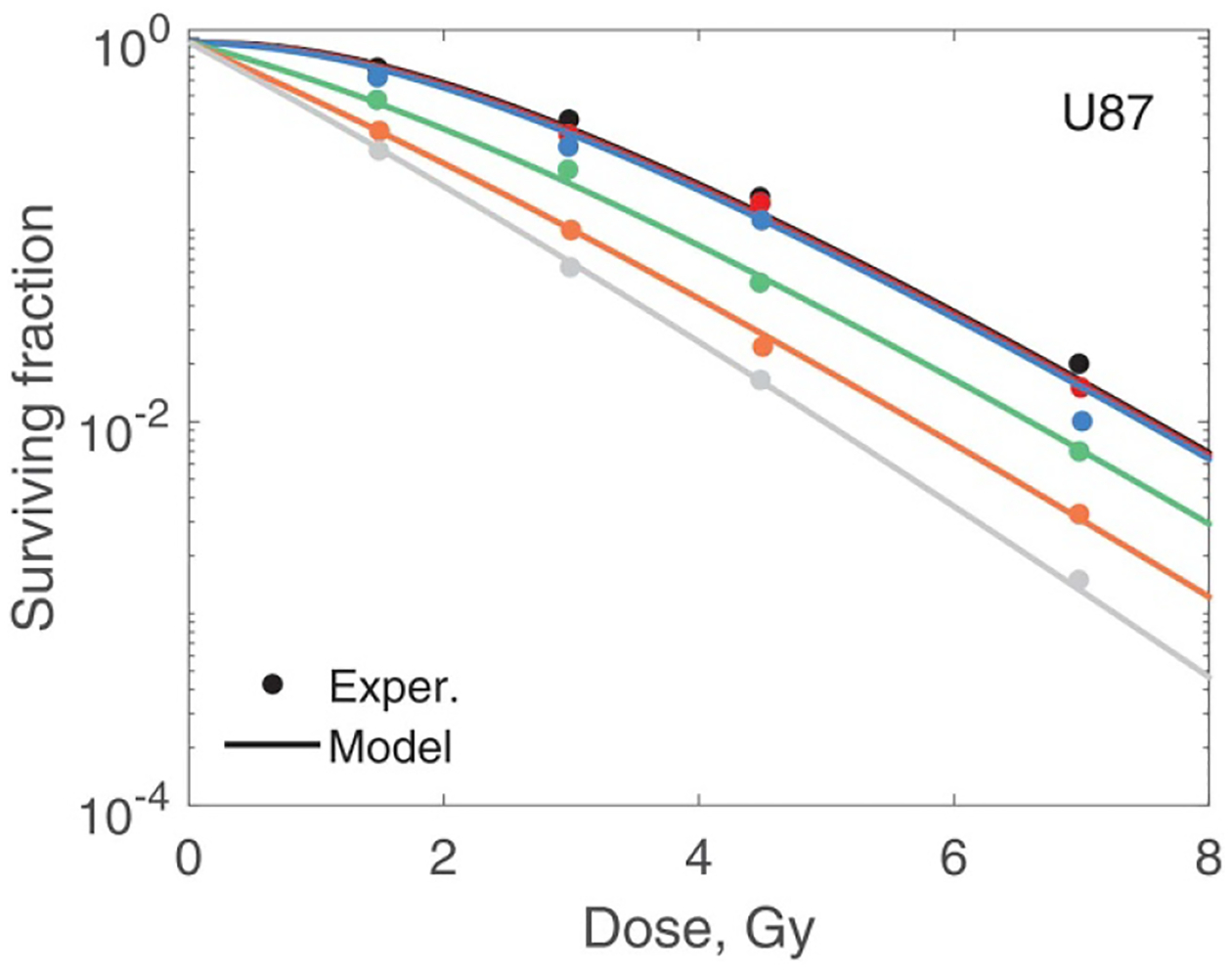
Modelling of U87 cell survival (Chaudhary *et al* 2014). Experiments 7–12 from table 1 are shown from top to bottom. Circles and lines of the same colour represent the same experiment. The overall r.m.s. error is 0.13 and adjusted $r^{2}$ is 0.99. Adapted from Chaudhary *et al* (2014), Copyright (2014), with permission from Elsevier.

**Figure 9.**
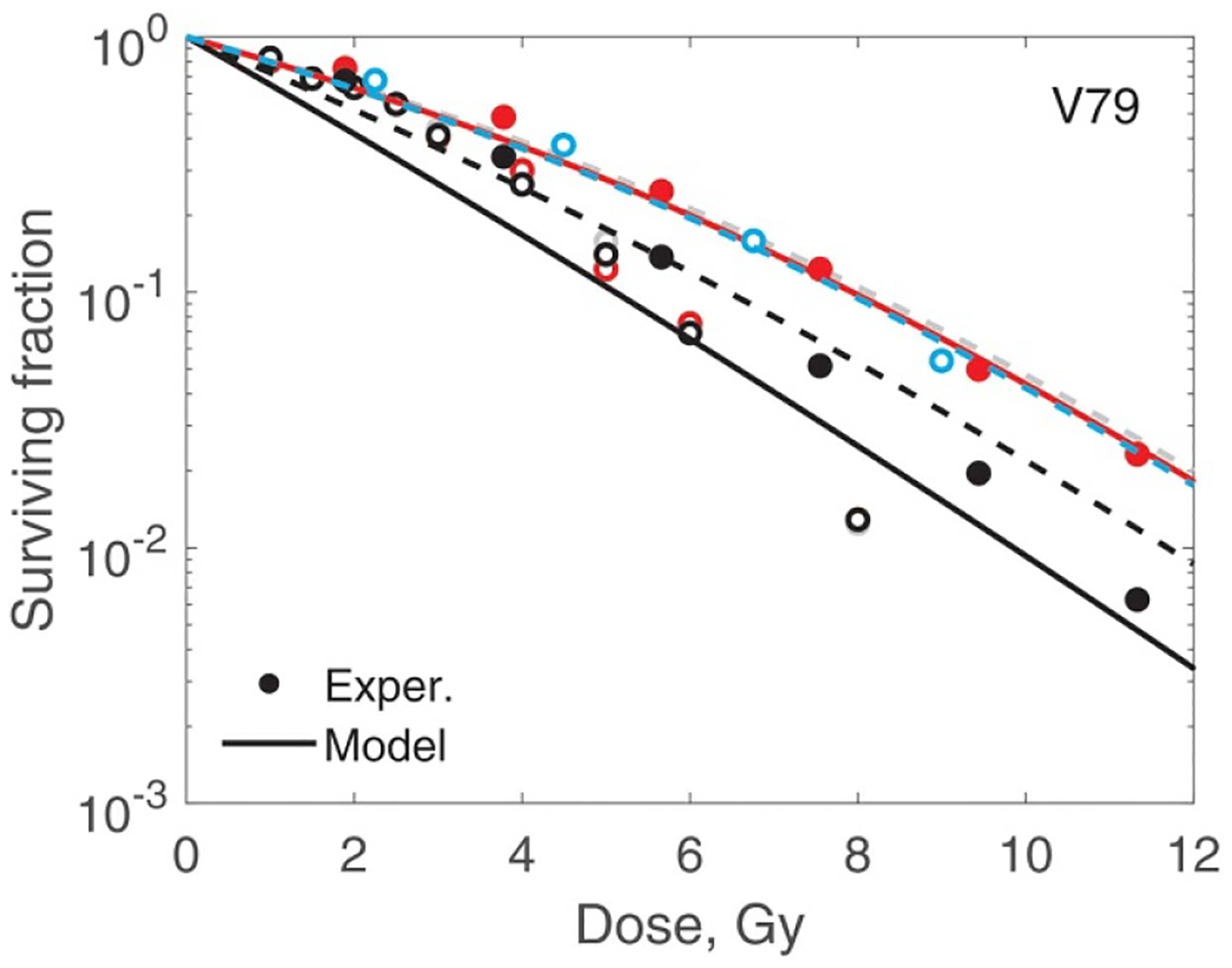
Modelling of V79 cell survival. Experiments are shown in which $D_{10}$ is in the range 6.33–8.72 Gy. Using numbering from table 1: 14, solid red line and filled red circles; 15, black solid line and black filled circles; 16, grey dashed line and open grey circles; 17, red dashed line and open red circles; 20, dashed black line and open black circles; 21, blue dashed line and open blue circles. The overall r.m.s. error for all V79 data is 0.59 and adjusted $r^{2}$ is 0.84.

**Figure 10.**
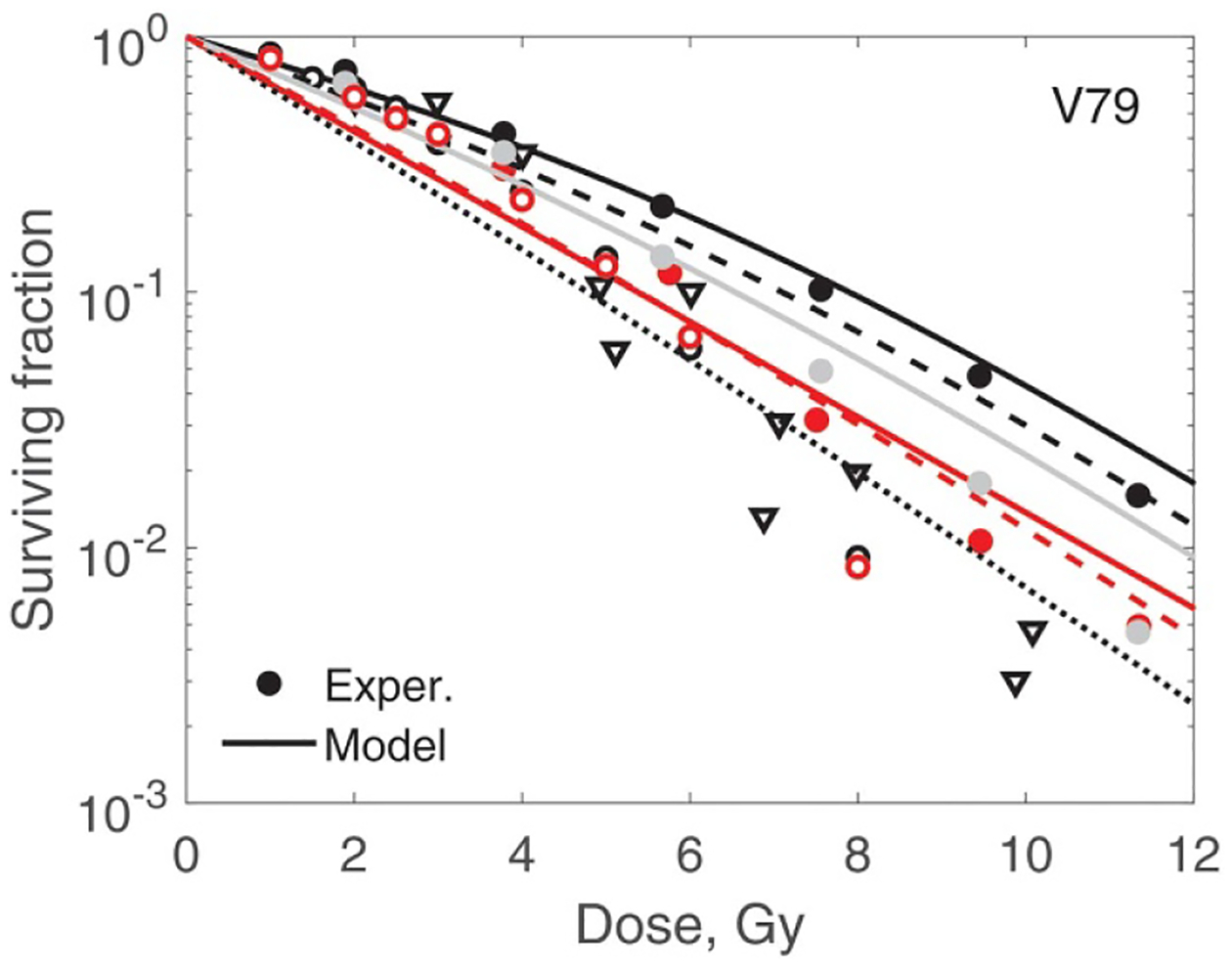
Modelling of V79 cell survival. Experiments are shown in which $D_{10}$ is in the range 5.26–6.10 Gy. Using numbering from table 1: 13, black dotted line and triangles; 18, dashed red line and open red circles; 19, solid red line and red filled circles; 22, dashed black line and open black circles; 23, solid black line and black filled circles; 24, solid grey line and grey filled circles. The overall r.m.s. error for all V79 data is 0.59 and adjusted $r^{2}$ is 0.84.

In figures 7–10 best-fit survival curves are compared with experimental data. Good agreement was achieved for AG01522 and U87 cells. These were single source data (Chaudhary *et al* 2014). In contrast, we selected V79 data from several indendent sources that used a variety of beam energies and SOBP parameters. As a result, we observed discrepancies, sometimes substantial, between different studies. We, however, consider this as a positive property of our V79 set. These discrepancies reflect uncertainties in experimental data of this type. We divided our V79 results into two subsets based on $D_{10}$ values (given in the captions). $D_{10}$ is the dose at which 10% of cells survive. These results are shown in figures 9 and 10. Our model correctly outlines variations of the data with dose and beam quality. However, accurate fit was not achieved for each individual curve.

**Figure 11.**
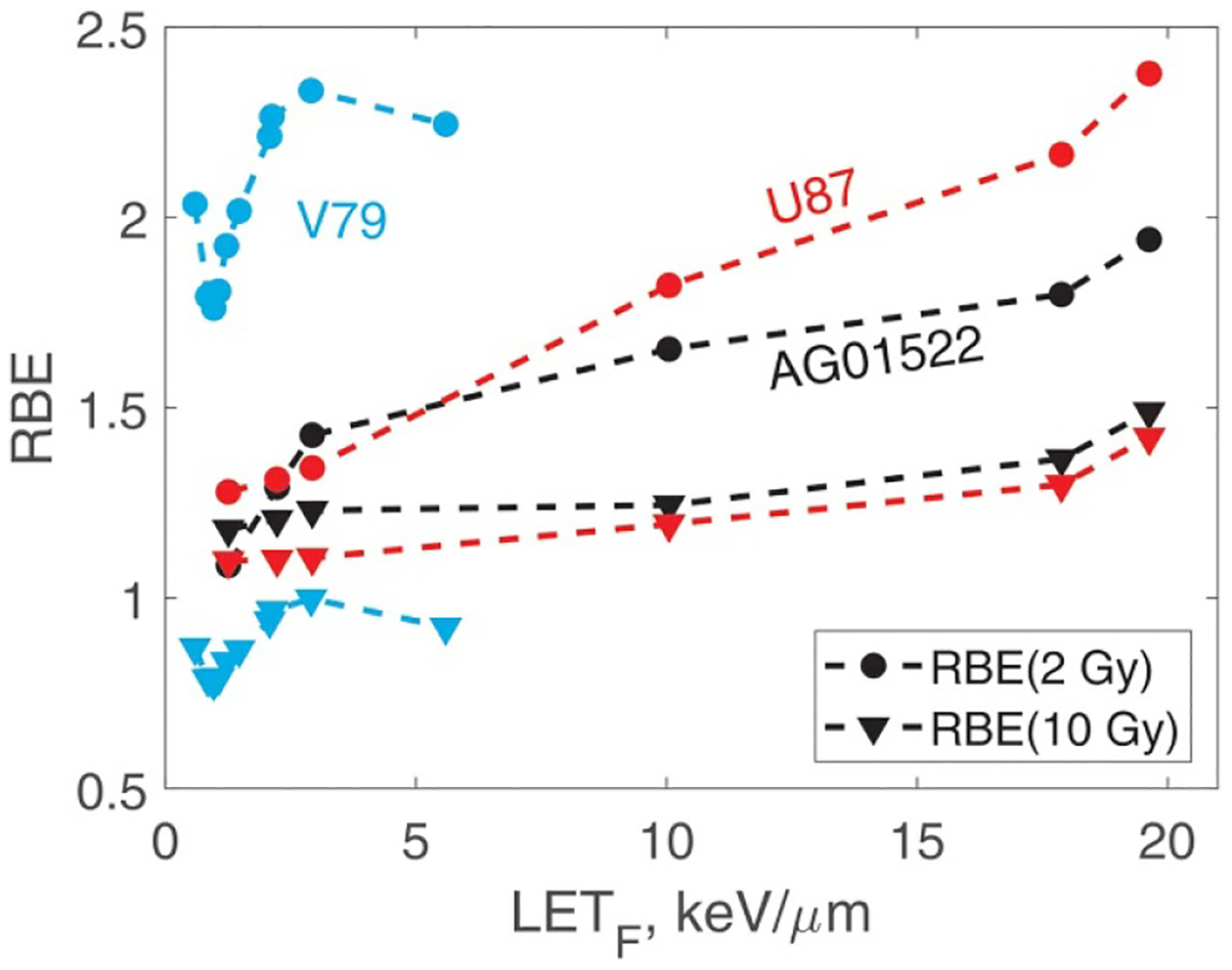
RBE at proton doses 2 Gy and 10 Gy versus LET_F_. Note that the reference radiation is 225 kVp x-rays for AG01522, and U87 cells and ^60^Co for V79 cells.

In figure 11, we show RBE as function of LET_F_ calculated from the best-fit survival curves. The RBEs were calculated at proton doses of 2 Gy and 10 Gy. To help understand this figure, we note that at 10 Gy, RBE is largely determined by $z_{\text{F}}$ values. At 2 Gy the damage repair ability (i.e. $s_{i}$) becomes a strong factor. A particularly interesting result is for V79 cells. In this case, the RBE at 2 Gy suggests a high probability of damage repair. However, at 10 Gy owing to a rather high $z_{\text{F}}$, the RBE decreases to values close to and below 1 (relative to ^60^Co). Please note that all V79 data are associated with high uncertainties. Table 2, figures 1, 9 and 10 give a good idea of their magnitude.

**Figure 12.**
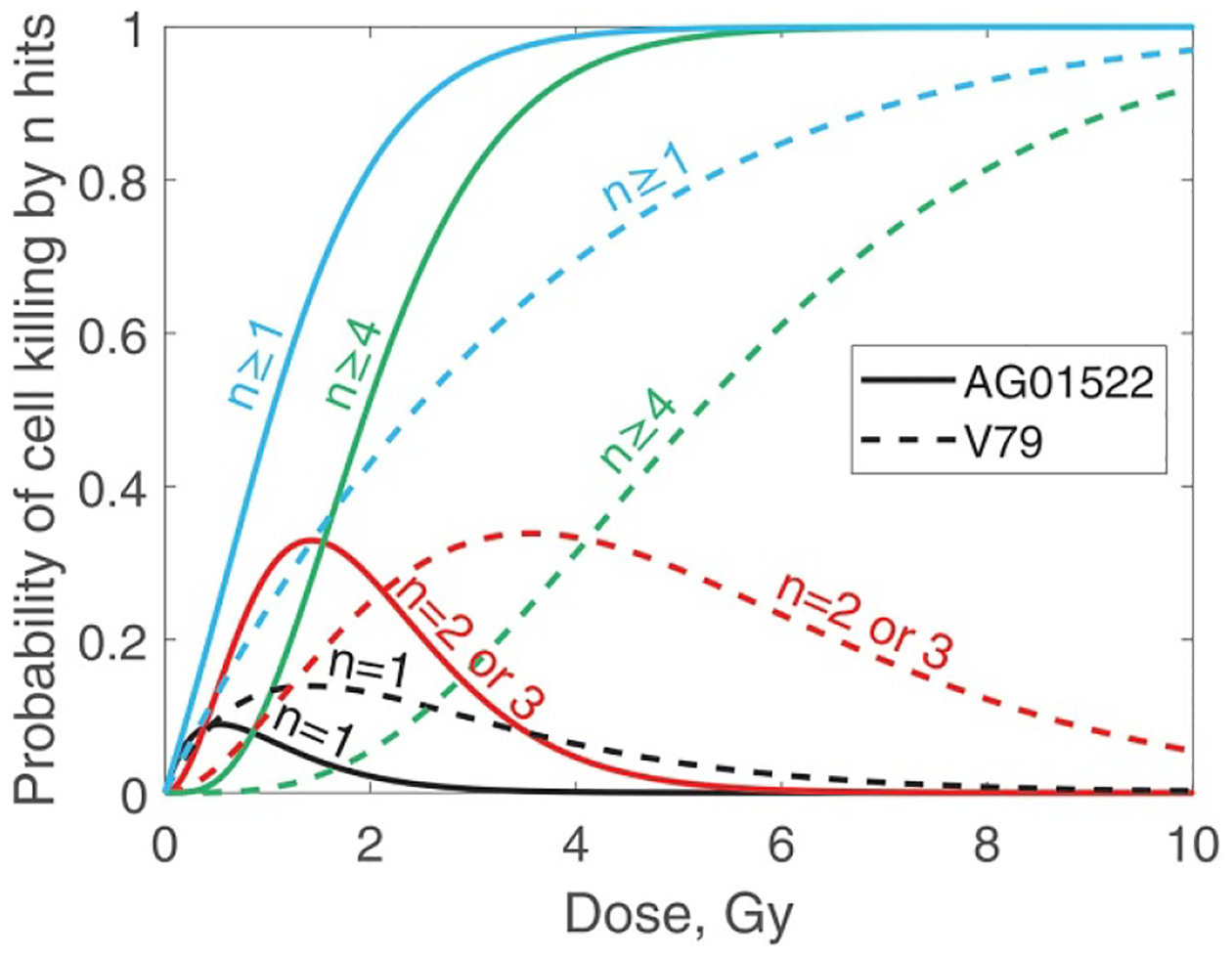
Probability of killing a cell by n protons entering the sensitive volume (n hits).

Finally, figure 12 summarises mechanisms of cell-kill by protons as a function of dose. This figure shows that the main mechanism of killing AG01522 cells at 2 Gy and 10 Gy involves four or more protons. For V79 cells the main mechanism at 2 Gy involves two or three protons, and more than three protons mechanism dominates at 10 Gy. The smaller numbers of protons for V79 cells can be attributed to a smaller SV size. The single-proton mechanism dominates below 0.38 Gy (AG01522) and 1.22 Gy (V79). These results have serious implications for now popular studies of DNA damage that use microscopic Monte Carlo simulations. In a typical setup, a single proton is launched and DNA damage is recorded. Then, before the next proton is launched, the DNA is reset to its original undamaged state. Such techniques can predict only a single-proton mechanism, which is a minor factor at therapeutic doses.

## 4. Conclusions

The present study addresses the need for a cell survival model that is accurate over a broad range of doses, including high doses used in hypofractionated radiotherapy and in radiobiological experiments. We combined two previously existing approaches: a non-LQ cell survival model that predicts the dose dependence and satisfies the above requirement, and the most general version of the microdosimetric approach for predicting beam quality dependence. This combination has eliminated two serious problems of the standard microdosimetry: arbitrary choice of SV size and the need to calculate dose-dependent multi-particle microdosimetric spectra f(q∣D). In our model, SV size is essentially a fitting parameter. We found that SV size increases with increasing LET. In the standard microdosimetry SV size is constant. Variable SV size reflects real physical, chemical and biological processes that cause spatial spread of radiation-induced damage. Damage spreads by up to several hundred nanometres from the particle path.

We have tested our model by applying it to a set of 24 proton survival curves for three cell lines. The set includes a variety of irradiation conditions and covers a broad LET range. We achieved good agreement between all these measurements and the model. Our analysis shows that the shape of survival curves is a result of an interplay between cell ability to repair damage and the extent of spatial spread of damage. At sufficiently high doses, the cell’s ability to repair damage becomes a negligible factor. Our data also show that at therapeutic doses cell kill results predominantly from a combined damage from several protons. The single-proton mechanism is a minor factor and becomes negligible above approximately 10 Gy.

Our model is relatively simple and suitable for implementation in a treatment planning system. This model has the potential to improve treatment plan optimisation, calculation of RBEs and biologically equivalent doses.

## Figures and Tables

**Table 1.** Cell survival data-set. The data are from four sources: Blomquist *et al* (1993), Robertson (1994), Chaudhary *et al* (2014), Wouters *et al* (2015). SOBP: spread-out Bragg peak.

	E(MeV)	SOBP (cm)	Depth (cm)	${LET}_{F}(keV\mu m^{-1})$	Cell type	Reference radiation	Reference
1	62	1.8–3.0	0.14	1.26	AG01522	225 kVp	Chaudhary
2	”	”	1.91	2.23	”	”	”
3	”	”	2.42	2.93	”	”	”
4	”	”	3.01	10.0	”	”	”
5	”	”	3.09	17.9	”	”	”
6	”	”	3.12	19.6	”	”	”
7	”	”	0.14	1.26	U87	”	”
8	”	”	1.91	2.23	”	”	”
9	”	”	2.42	2.93	”	”	”
10	”	”	3.01	10.0	”	”	”
11	”	”	3.09	17.9	”	”	”
12	”	”	3.12	19.6	”	”	”
13	67	1.3–3.3	2.2	2.91	V79	^60^Co	Blomquist
14	155	5.3–13.2	4.5	0.844	”	”	Robertson
15	”	”	12.2	2.12	”	”	”
16	160	6–16	8.4	0.964	”	”	Wouters
17	”	”	9.7	1.06	”	”	”
18	”	”	14.7	2.08	”	”	”
19	200	13.0–21.1	21	5.59	”	”	Robertson
20	230	19–29	13.5	0.589	”	”	Wouters
21	”	”	24.4	1.01	”	”	”
22	”	”	26.1	1.22	”	”	”
23	250	23.1–31.4	23.5	0.898	”	”	Robertson
24	”	”	28.8	1.47	”	”	”

**Table 2.** Cell survival parameters for photons. r.m.s.: root mean square error.

Cell type, radiation	$z_{F}$, Gy	$s_{i}$				$lnS_{0}$	r.m.s. error	Adjusted $r^{2}$
AG01522,		i=0	i=1	i=2	i>2			
225 kVp	0.6749	1	0.8568	0.1289	0	−0.05	0.0619	0.998
U87,		i<3	i=3	i=4	i>4			
225 kVp	0.8592	1	0.6245	0.08 200	0	−0.05	0.0839	0.998
V79,		i<5	i=5	i=6	i>6			
^60^Co	0.6901	1	0.8383	0.5100	0	−0.05	0.667	0.919

**Table 3.** Polynomial coefficients of the regression model equation (14) for $B_{z}(E)$.

Cell type	$p_{1},{Gy}^{-1}$	$p_{2},{Gy}^{-1}MeV$	$p_{3},{Gy}^{-1}{MeV}^{2}$	$p_{4},{Gy}^{-1}{MeV}^{3}$
AG01522	0.022 3367	−0.311 991	−1.940 73	1.898 44
U87	0.020 6750	−0.492 401	0.000 763 355	1.097 62
V79	−0.115 889	−8.314 79	−0.003 748 09	0.711 743

**Table 4.** Best-fit parameters for the biological response function B(q) defined in equations (2) and (15).

Cell type	b,keV	c	$q_{0},keV$	$lnS_{0}$
AG01522	1.427 99	0.953 716	−0.047 1277	−0.05
U87	19.6033	0.999 835	−0.002 238 96	−0.05
V79	0.504 724	1.515 47	0.137 499	0.05

## Data Availability

The data cannot be made publicly available upon publication because no suitable repository exists for hosting data in this field of study. The data that support the findings of this study are available upon reasonable request from the authors.
